# LC-ESI-QTOF/MS Characterization of Phenolic Compounds in Palm Fruits (Jelly and Fishtail Palm) and Their Potential Antioxidant Activities

**DOI:** 10.3390/antiox8100483

**Published:** 2019-10-14

**Authors:** Chao Ma, Frank R. Dunshea, Hafiz A. R. Suleria

**Affiliations:** School of Agriculture and Food, Faculty of Veterinary and Agricultural Sciences, The University of Melbourne, Parkville, VIC 3010, Australia; chaom2@student.unimelb.edu.au (C.M.); fdunshea@unimelb.edu.au (F.R.D.)

**Keywords:** jelly palm, fishtail palm, polyphenols, flavonoid, phenolic acids, antioxidant activity, HPLC-PDA, LC-ESI-QTOF/MS

## Abstract

Palm fruits have gained growing attention for their nutrition values and health promotion perspectives. They have a diverse range of bioactive compounds including carotenoids, vitamins, dietary fibres and especially polyphenolic compounds. These polyphenolic compounds contribute to the putative health benefits of palm fruits. Nevertheless, the detailed information about these polyphenols in palm fruits is limited. The present work was conducted to comprehensively characterize polyphenols in two palm fruits, jelly palm (*Butia ordorata*) and fishtail palm (*Caryota uren*), using liquid chromatography electrospray ionization quadrupole time-of-flight mass spectrometry (LC-ESI-QTOF/MS) and assess their antioxidant potential. The total phenolic content (TPC), total tannins content (TTC), 2,2-diphenyl-1-picrylhydrazyl (DPPH) antioxidant assay and 2,2′-azinobis-(3-ethylbenzo-thiazoline-6-sulfonic acid) (ABTS) scavenging abilities and ferric reducing antioxidant power (FRAP) were higher in the jelly palm fruit while total flavonoid contents (TFC) were higher in the fishtail palm. The LC-ESI-QTOF/MS tentatively identified a total of 86 phenolic compounds in both jelly and fishtail palm fruits. Although both palm fruits exhibited different phenolic profiles, hydroxycinnamic acids and flavonols were the most common in both. In high performance liquid chromatography photodiode array (HPLC-PDA) quantification, 4-hydroxybenzoic acid (317.46 ± 4.68 µg/g) and catechin (4724.00 ± 32.39 µg/g) were the most abundant phenolic acid and flavonoid quantified in the jelly palm fruit, respectively. Quercetin (557.28 ± 7.81 µg/g) and kaempferol 3-*O*-glucoside (220.99 ± 2.06 µg/g) were the most abundant flavonoids quantified in the fishtail palm. Our study indicates that palm fruit is a good source of polyphenols and has strong antioxidant potential for health promotion. Furthermore, this study provides the scientific basis for an exploitation of jelly and fishtail palm fruits in the food, pharmaceutical and nutraceutical industries.

## 1. Introduction

Palm fruits are mostly grown in tropical regions and are rich sources of bioactive compounds such as polyphenols, vitamin E, carotenoids and unsaturated fatty acids [1]. Bioactive compounds extracted from different palm fruits are reported as being effective against various disorders including ageing, cancer, cardiovascular disease, nerve dysfunction, respiratory distress syndrome and diabetes [2]. Thus, the bioactive compound extracts from different palm fruits, especially polyphenols, may contribute to human health. 

Jelly palm (*Butia ordorata*) is a tropical palm species whose fruits and leaves can be processed into different food products [3]. The jelly palm fruit is mainly consumed fresh or used as an ingredient in pulp, juices, alcoholic beverages and frozen products [4]. The pulp and leaves of the *Butia* family were also used for treatment of skin diseases and venom infection [5] because of the presence of diverse bioactive compounds. Jelly palm contains various bioactive compounds including polyphenols, carotenoids and dietary fibres. Among polyphenols, hydroxybenzoic acid and catechins have been detected in abundance in different genotypes of jelly palm [6]. Fishtail palm (*Caryota uren*) is also one of the most common subspecies of palm and is distributed widely in Asia. The sap of fishtail palm can be processed into sugar or used to make palm wine [7]. The honey and jaggery from fishtail palm can be used as sweeteners in different food industries. The bark is usually used for treatment of rheumatic disease and snake poisoning [8], which may suggest the antioxidant potential of the fishtail palm fruit. The antioxidant and antimicrobial potential of fishtail palm fruits were reported by Ananth et al. [9]. Some polyphenols, including flavonoids and coumarins, were identified and quantified in fishtail palm [10]. Polyphenols including yellow flavonoids, procyanidins and cyanidin-3-*O*-glucoside in substantial amounts have also been characterized in different palm fruits like juçara (*Euterpe edulis*), patawa (*Oenocarpus bataua*) and açai (*Euterpe oleracea*) palm fruit [1].

Polyphenols are secondary metabolites from plants and many have antioxidant activities [11]. They can be extracted using different organic solvents while their antioxidant potential can vary depending upon the type of extraction, conditions and the choice of solvents [12]. The antioxidant activity of polyphenolic compounds is evaluated using different in vitro spectrophotometric-based assays to analyse the capacities of electron or free radical scavenging of plant material with specific mechanisms. Total phenolic content (TPC), total flavonoid content (TFC), total tannin content (TTC), radical scavenging activity using 2,2-diphenyl-1-picrylhydrazyl (DPPH) antioxidant assay, 2,2′-azinobis-(3-ethylbenzothiazoline-6-sulfonic acid) (ABTS) assay and reducing ability using ferric reducing antioxidant potential (FRAP) assays are used to map overall antioxidant potential.

Polyphenols can be separated, characterized and quantified using high performance liquid chromatography (HPLC) and liquid chromatography-mass spectrometry (LC-MS). The LC-electrospray ionization (ESI) quadrupole time-of-flight (QTOF) mass spectrometry (MS) is effective in characterizing and quantifying the complex phenolic compounds and other metabolites. Previously, only a few studies have been done to provide chemical characterization of jelly palm and fishtail palm, with limited research being conducted on the extraction, isolation and characterization of polyphenols from these palm fruits. In this study, we extracted polyphenols from jelly and fishtail palm fruits and analysed their antioxidant potential. Moreover, identification, characterization and quantification of specific phenolic constituents were achieved through LC-ESI-QTOF/MS and HPLC photodiode array (PDA). This article is an effort to provide more reliable information about the antioxidant and beneficial health properties of palm fruits in order to promote their use in different food, pharmaceutical and supplement industries.

## 2. Materials and Methods 

### 2.1. Chemicals and Reagents 

Most of the chemicals used for extraction and characterization were analytical grade and purchased from Sigma-Aldrich (Castle Hill, NSW, Australia). The standards for antioxidant assays included gallic acid, quercetin, catechin and L-ascorbic acid, which were purchased from Sigma-Aldrich (St. Louis, MO, USA). For antioxidant assays, Folin and Ciocalteu’s phenol, aluminium chloride hexahydrate, vanillin, 2,2-diphenyl-1-picrylhydrazyl (DPPH), ferric (III) chloride anhydrous, 2,4,6-tripyridyl-s-triazine (TPTZ), potassium persulfate and 2,2′-azino-bis(3-ethylbenz-thiazoline-6-sulphonate) (ABTS) were purchased from Sigma-Aldrich (St. Louis, MO, USA) and sodium carbonate anhydrous (chem-supply; Gillman, SA, Australia), sodium acetate hydrated (Ajax Finechem, Scoreby, VIC, Australia) and sulfuric acid 98% were purchased from RCI Labscan Limited, (Bangkok, Thailand). The HPLC grade methanol was purchased from Fisher Chemical Company (San Jose, CA, USA). The mobile phases for LC-ESI-QTOF/MS and HPLC were mainly comprised of 50% acetic acid solution (Sigma-Aldrich, St. Louis, MO, USA) and acetonitrile (LiChrosolv, Darmstadt, Germany). The reference standards including protocatechuic acid, catechin, 4-hydroxybenzoic acid, chlorogenic acid, quercetin 3-O-glucoside, kaempferol 3-O-glucoside and quercetin were purchased from Sigma-Aldrich (St. Louis, MO, USA).

### 2.2. Sample Preparation

Samples used for this study were ripened jelly palm (*B. ordorata*) and fishtail palm (*C. uren*) fruits, harvested directly from palm trees in Geelong, Victoria, Australia. The fresh palm fruits were collected and weighed for 2–3 kg; jelly palm fruits were blended into a slurry using a 1.5 L blender (Russell Hobbs Classic, model DZ-1613, Melbourne, VIC, Australia) while the fishtail palm fruits were grounded, and the pericarp was collected. Samples were kept at −20 °C for further analysis. 

### 2.3. Extraction of Phenolic Compounds

Extracts were prepared by homogenizing with the Ultra-Turrax T25 Homogenizer (IKA, Staufen, Germany) in 30% (*v/v*) ethanol at 10,000 rpm for 30 s followed by incubation in a ZWYR-240 incubator shaker (Labwit, Ashwood, Vic, Australia) at 120 rpm at 4 °C for 12 h. After incubation, all samples were centrifuged using the Hettich Refrigerated Centrifuge (ROTINA 380R, Tuttlingen, Baden-Württemberg, Germany) at 5000 rpm for 15 min. The supernatant was collected and diluted with ethanol at appropriate ratios for the various antioxidant analysis. 

### 2.4. Antioxidant Assays 

All the antioxidant assays were performed by adopting the method of Gu et al. [13]. The data was measured by the Multiskan^®^ Go microplate photometer (Thermo Fisher Scientific, Waltham, MA, USA). All tests were run in triplicate. The standard curves were created with *R*^2^ > 0.995.

#### 2.4.1. Determination of Total Phenolic Content (TPC)

The TPC in palm samples was determined by modifying the method of Severo et al. [14]. In total, 25 µL of 25% (*v/v*) Folin–Ciocalteu reagent and 200 µL Milli-Q water were added to 25 µL of extracts in triplicates in 96-well plates (Costar, Corning, NY, USA) and incubated for 5 min at room temperature. Then, 25 µL of 10% (*w/w*) sodium carbonate was added into the reaction mixture and kept in a dark room for 1 h at room temperature. Absorbance was measured at 764 nm in a plate reader. The absorbance was converted to total polyphenol content based on the calibration curve prepared by the gallic acid standard with concentration ranging from 0 to 200 µg/mL. The TPC was expressed as mg of gallic acid equivalents per gram of the sample (mg GAE/g of raw material) on the basis of fresh weight (_fw_).

#### 2.4.2. Determination of Total Flavonoids Content (TFC) 

The TFC of palm fruits was evaluated by modifying the aluminium chloride method of Gouveia and Castilho [15]. In total, 80 µL of the diluted sample was mixed with 80 µL of 2% (*w/v*) aluminium chloride ethanolic solution and 120 µL of 50 mg/mL sodium acetate. Then, the absorbance of the reaction mixture was measured at 440 nm after 1 h incubation in a dark room at room temperature. The values of TFC expressed in quercetin equivalent (µg QE/g _fw_) were calculated from the standard curve (quercetin: 0 to 50 µg/mL).

#### 2.4.3. Determination of Total Tannins Content (TTC)

The TTC was estimated by modifying the method of Zou et al. [16]. In total, a 25 µL sample was added in 150 µL of 4% (*w/v*) methanolic vanillin solution. Then, 25 µL of 32% (*v/v*) sulfuric acid in methanol was added in the solution mixture and incubated at 25 °C for 15 min. The absorbance was measured at 500 nm and the tannins in samples were quantified by linear regression plotting the absorbance against standard catechin concentration (0–1000 µg/mL). The absorbance was converted to concentration of tannins with the unit of mg of catechin equivalent per gram of sample (mg CE/g).

#### 2.4.4. 2,2-Diphenyl-1-picrylhydrazyl (DPPH) Antioxidant Assay

The DPPH free radical scavenging capacity was calculated by modifying the method of Alvarez-Jubete et al. [17]. In total, 260 µL of 0.1 mM of DPPH radical methanol solution was added into 40 µL samples. The absorbance of the reaction mixture was measured at 517 nm after incubating for 30 min. The scavenging activity against DPPH free radicals was expressed as units of ascorbic acid equivalent (mg AAE/g _fw_) calculated based on the calibration curve constructed with a standard ascorbic acid solution ranging from 0 to 50 µg/mL.

#### 2.4.5. Ferric Reducing Antioxidant Power (FRAP) Assay

The FRAP method evaluates the capacity of a substance to reduce Fe^3+^ in the Fe^3+^-TPTZ complex (ferric-2,4,6-tripyridyl-s-Triazine) into Fe^2+^-TPTZ. The reducing antioxidant power of samples was estimated by modifying the method of Chen et al. [18]. The FRAP reagent was prepared freshly by adding 20 mM FeCl_3_ and TPTZ solution (10 mM TPTZ and 40 mM HCl) into 300 mM sodium acetate solution with the volume ratio of 1:1:10. Then, 280 µL of FRAP reagent was mixed with 20 µL of extract. The reaction mixture was incubated at 37 °C for 10 min and absorbance was measured at 593 nm. Concentrations of 0 to 50 µg/mL of ascorbic acid were prepared to construct the standard curve. The results were expressed as mg ascorbic acid equivalents per g of fresh sample weight (mg AAE/g _fw_). 

#### 2.4.6. 2,2’-Azinobis-(3-ethylbenzo-thiazoline-6-sulfonic acid) (ABTS) Radical Scavenging Assay

Free radical scavenging activity was measured by modifying the method of Severo, Tiecher, Chaves, Silva and Rombaldi [14]. The free radical ABTS cations are generated after mixing 7 mM ABTS and 140 mM potassium persulfate (volume ratio: 625:11) followed by incubation in the dark for 16 h. The stock solution was further diluted with ethanol to obtain the ABTS cation solution with an absorbance range of 0.70 ± 0.02 at 734 nm. Then, 290 µL ABTS cation solution was mixed with 10 µL sample. The absorbance was measured at 734 nm after incubation at room temperature for 6 min. The results were expressed as mg ascorbic acid equivalent per gram of sample (mg AAE/g _fw_). 

### 2.5. LC-ESI-QTOF/MS Characterization of Phenolic Compounds

The LC-ESI-QTOF/MS analysis was carried out by modifying the method of Mateos-Martin et al. [19]. Agilent 1200 series HPLC (Agilent Technologies, Santa Clara, CA, USA) equipped with Agilent 6520 I Accurate-Mass Q-TOF LC/MS (Agilent Technologies, Santa Clara, CA, USA) was used for the identification of polyphenolic compounds. The separation of each compound was carried out using Synergi Hydro-RP 80A LC reverse phase column with an internal diameter of 250 mm × 4.6 nm and particle size of 4 µm (Phenomenex, Lane Cove, NSW, Australia). The column was protected by the Phenomenex C18 ODS guard column with an internal diameter of 4.0 × 2.0 mm. The mobile phase A was acetic acid/water solution (2:98 *v/v*) and mobile phase B was acetonitrile/water/acetic acid (100:1:99, *v/v/v*). In total, 6 µL of samples were injected and the separation followed a gradient programme with flow rate of 0.8 mL/min for an 85-min run where the ratio of mobile phase B changed from 10% to 25% in 20 min, from 25% to 35% in 10 min, from 35% to 40% in 10 min, from 40% to 55% in 30 min, from 55% to 80% in 5 min and from 80% to 100% in 2 min followed by maintenance for 2 min. The ratio of mobile phase B was adjusted to 10% from 100% in 3 min after the whole separation process and kept isocratic for 3 min. The samples and column were maintained at 10 °C and room temperature, respectively. The pressure of nitrogen gas condition was set at 45 psi at 300 °C with a flow rate of 5 L/min, while the parameter of sheath gas was set with a flow rate of 11 L/min at 250 °C. The capillary and nozzle voltage were set at 3.5 kV and 500 V, respectively. The complete mass scan was in the range of *m/z* 50–1300. The control of the process, data collection and identification of phenolic compounds was performed on MassHunter workstation software (Qualitative Analysis, version B.03.01, Agilent). The LC-ESI-QTOF/MS identified compounds with more than 80 library identification scores were further selected for characterization and *m/z* verification.

### 2.6. HPLC-PDA Analysis

The quantification of targeted phenolic compounds present in palm samples was carried out by Agilent 1200 series HPLC (Agilent Technologies, CA, USA) equipped with a photodiode array (PDA) detector. The same column and conditions were maintained as described above in LC-ESI-QTOF/MS except for sample injection volume of 20 μL. The compositions of extracts were detected under λ 280 nm, 320 nm, and 370 nm by PDA detector. The individual polyphenol was quantified based on linear regression of external standards plotting peak area against concentration. Data acquisition and analysis were performed using Agilent LC-ESI-QTOF/MS MassHunter workstation software (Qualitative Analysis, version B.03.01, Agilent). 

### 2.7. Statistics Analysis

All the analyses were performed in triplicate. The values were expressed as mean ± standard deviation (SD). One-way analysis of variance (ANOVA) by Tukey’s test was carried out using Minitab^®^ 18 Statistical software (Minitab Inc., State College, PA, USA) for comparisons of antioxidant activities and the polyphenol contents between samples and *p* < 0.05 was considered statistically significant. 

## 3. Results and Discussion

### 3.1. Polyphenol Estimation (TPC, TFC and TTC)

Jelly and fishtail palm fruits have been reported as good sources of polyphenols [6,20]. The polyphenol contents in ethanolic extracts of jelly and fishtail palm fruits were characterized by TPC, TFC and TTC. The extract of jelly palm fruit was higher in polyphenols (3.74 ± 0.10 mg GAE/g _fw_) as compared to fishtail palm (1.75 ± 0.02 mg GAE/g _fw_) at *p* < 0.05 (Table 1). The TPC of jelly palm in our study was higher than methanolic extracts of the same species (1.71 mg GAE/g _fw_) reported by Hoffmann et al. [21], while Krishnamoorthy, Senguttuvan and Krishnaswamy [10] found lower TPC (0.9 mg GAE/g) in the ethanolic extract of fishtail palm. Our total polyphenols content in the jelly palm is comparable to five other genotypes of jelly palms from Brazil (2.65 to 4.02 mg GAE/g) [6]. Jachna et al. [22] also demonstrated that cold storage and pasteurization did not affect the total polyphenolic content in the jelly palm. This might be an advantage for the potential application of jelly palm polyphenols in food, feed and pharmaceutical industries. 

Flavonoids are a large class of polyphenols with diverse structures, which have gained a lot of attention due to their potent antioxidant and possible anticancer activities. Some subclasses of flavonoids had been involved in clinical trials [23]. Thus, the flavonoid content is an important index for nutritional assessment in food ingredients. The TFC of jelly palm (63.11 ± 0.51 µg QE/g _fw_) was significantly lower as compared to fishtail palm (207.80 ± 4.8µg QE/g _fw_) at *p* < 0.05 (Table 1). Previously, Sujitha and Kripa [20] measured TFC in aqueous and 70% ethanolic extracts of fishtail palm and found higher values than reported in our study. The variation of TFC might be because of the choice of extracts solvents and concentration. Regarding the TTC, the jelly palm had a higher level of TTC (4.06 ± 0.04 mg CE/g) as compared to fishtail palm (1.02 ± 0.01 mg CE/g), as indicated in Table 1. Krishnamoorthy, Senguttuvan and Krishnaswamy [10] reported that the TTC of fishtail palm fruits ranged from 0.5 to 1.6 mg GAE/g. Our study was consistent with some of the previous findings that indicated jelly and fishtail palm fruits are a great source of polyphenols. 

### 3.2. Antioxidant Activities (DPPH, FRAP and ABTS) 

Different methods are used for determining the antioxidant capacity of food materials. In this study, DPPH, FRAP and ABTS assays were used to quantify the free radical scavenging activity of jelly and fishtail palm fruits. The results of three assays were expressed as mg ascorbic acid/g _fw_ (mg AAE/g _fw_). Table 1 shows that the jelly palm exhibits higher DPPH free radical scavenging activity (2.58 mg ± 0.06 AAE/g) as compared to fishtail palm (0.31 ± 0.01 mg AAE/g) at *p* < 0.05. Sganzerla [24] reported DPPH activity in jelly palm, and found a positive correlation between antioxidant activity and phenolic contents. Fonseca [25] also found DPPH antioxidant activity of jelly palm ranging from 828.27 to 1295.25 mg Trolox equivalent per gram of fruit.

The antioxidant capacity of palm fruits was also determined by the capacity to reduce Fe(III). The FRAP antioxidant activity of jelly palm and fishtail palm was 1.29 ± 0.01 mg AAE/g and 0.03 ± 0.01 µg AAE/g, respectively (*p* < 0.05). Previously, Denardi, et al. [26] reported higher FRAP activity in another subspecies of jelly palm (*Butia eriospatha*). Chen et al. [18] also observed higher FRAP activity in freeze-dried proanthocyanidins extracts of another species of fishtail palm (*Caryota ochlandra*). The ABTS free radical scavenging activity expressed as mg ascorbic acid/g _fw_ was measured by a decolorization assay. Similar to the TPC, DPPH and FRAP assays, jelly palm exhibits higher ABTS (4.38 ± 0.05 mg AAE/g) as compared to fishtail palm (1.85 ± 0.04 mg AAE/g). The ABTS free radical scavenging activity of our jelly palm is comparable to jelly palms (average 4.05 mg Trolox equivalent antioxidant capacity/g) in Brazil [18]. 

In our study, the estimated antioxidant activity (TPC, DPPH, FRAP and ABTS) in the jelly palm was higher than the fishtail palm. Similarly, Hoffmann, Carvalho, Barbieri, Rombaldi and Chaves [21] reported the correlation between DPPH antioxidant activity and TPC (*r^2^* = 0.64). Thus, the antioxidant capacity of jelly and fishtail palm fruits can be associated with their polyphenol content. Variations in antioxidant activity in palm fruits can be due to diverse polyphenol ranges in different species, subspecies, genotypes and varieties cultivated at different environments [17]. Also, the type of solvents and extraction methods can impact on polyphenol profile. Boeing et al. [27] found significant differences in polyphenol content, antioxidant activity and amount of particular phenolic compounds in different solvent extracts. In this study, both qualitative and quantitative analyses were carried out to further investigate the presence of actual phenolic components in both jelly and fishtail palm fruits. For the reason, LC-ESI-QTOF/MS and HPLC-PDA were used for identification, characterization and quantification of polyphenols in palm samples.

### 3.3. LC-ESI-QTOF/MS Characterization of Phenolic Compounds

Untargeted qualitative analysis of phenolic compounds in jelly and fishtail palm fruit was performed on LC-ESC-QTOF/MS. Polyphenols were tentatively identified and characterized based on their *m/z* value from MS spectra in both positive and negative ionization modes (Figures S1 and S2). Table 2 and Table 3 show the lists of all the compounds tentatively identified in both palm fruits on the basis of their *m/z* value from MS spectra in both negative and positive ionization mode ([M − H]^−^/[M + H]^+^), using an Agilent LC/MS MassHunter Qualitative Software and Personal Compound Database and Library (PCDL) with online database. Compounds with a score more than 80 (PCDL score) and mass error <±10 ppm were only selected for characterization and *m/z* verification purposes. A total of 86 phenolic compounds were reported in jelly and fishtail palm fruits (Table 2 and Table 3); they were mainly flavonoids and phenolic acids followed by lignans, stilbenes and other polyphenols. A total of 41 flavonoids and 29 phenolic acids belonging to various polyphenol subclasses were characterized including hydroxycinnamic acids, hydroxybenzoic acids, flavonols, flavones, flavanones, flavanols and anthocyanins, etc. Two palm fruits showed the presence of different polyphenolic compounds, which leads to variation in TPC, TFC, TTC and overall antioxidant activity.

#### 3.3.1. Phenolic Acids

In our study, five different sub-classes of phenolic acids were tentatively characterized in jelly and fishtail palm fruits as shown in Table 2 and Table 3, which mainly consist of hydroxycinnamic and hydroxybenzoic acid derivatives. Hydroxyphenylacetic acids, hydroxyphenylpentanoic acids and hydroxyphenylpropanoic acids were also detected in both palm fruits. 

##### Hydroxycinnamic Acid

Compound 2 and 5 (retention time (RT) = 19.734 and 37.691 min) with [M − H]^−^ ion at *m/z* 353.0890 and 515.1187 detected in the ethanolic extract of jelly palm fruits were tentatively characterized as chlorogenic acid and 1,5-dicaffeoylquinic acid, respectively (Table 2), while chlorogenic acid in fishtail palm was detected in positive ionization mode with [M + H]^+^ at *m/z* 355.1017 (Table 3). There were five ferulic acid derivatives (compound 4, 5, 7, 8 and 9) found in fishtail palm fruits including ferulic acid 4-*O*-glucoside (RT = 25.81 min with *m/z* 355.1049), 3-feruloylquinic acid (RT = 30.1 min with *m/z* 367.1040), aglycones ferulic acid (RT = 37.919 min with *m/z* 193.0504), 1-sinapoyl-2-feruloylgentiobiose (RT = 41.78 min with *m/z* 723.2167) and 1,5-diferuloylquinic acid (RT = 63.86 min with *m/z* 543.1529) in negative ionization mode (Table 3), while ferulic acid 4-*O*-glucoside and 1-sinapoyl-2-feruloylgentiobiose with [M − H]^−^ ion at *m/z* 355.1049 and 723.2137 were detected in jelly palm fruit (Table 2). Sinapine characterized by [M − H]^−^ ion at *m/z* 309.1568 was proposed for fishtail palm. Previously, chlorogenic acid and aglycones of these compounds (caffeic acid, sinapic acid and ferulic acid) have already been detected in different solvent extracts of jelly palms [3,6,27]. The ferulic acid constituents in fishtail palm fruits have been characterized by LC-MS in the study of Sujitha and Kripa [20]. The presence of caffeic, sinapic and ferulic acid derivatives has already been reported in edible parts of açai and date palm fruits [28,29,30]. Cinnamic acid exhibited [M + H]^+^ ion at *m/z* 149.0585 and 149.0582 were detected in both palm fruits, respectively. Other hydroxycinnamic acids including 3-*p*-coumaroylquinic acid ([M + H]^+^ *m/z* 339.1081) and 3-*O*-methylrosmarinic acid ([M + H]^+^ *m/z* 375.1069) were tentatively characterized in fishtail palm. Hoffman et al. [3] discovered the aglycone of 3-*p*-coumaroylquinic acid (p-coumaric acid) in the methanolic extract of jelly palm pulp and nectar.

##### Hydroxybenzoic Acids

Two 4-hydroxybenzoic acid derivatives were tentatively characterized including aglycone 4-hydroxybenzoic acid with RT = 25.849 min and [M + H]^+^ at *m/z* 139.0394 and 4-hydroxybenzoic acid 4-*O*-glucoside with RT = 7.456 min and [M − H]^−^ at *m/z* 299.0793 in extracts of jelly and fishtail palm, respectively (Table 2 and Table 3). Hydroxybenzoic acid (*m/z* 137.0242) and hydroxybenzoic hexose (*m/z* 299.0070) have already been characterized in jelly palm by Hoffmann, Carvalho, Barbieri, Rombaldi and Chaves [21]. The NaOH hydrolysed sugar date palm fruits also showed the presence of hydroxybenzoic acid derivatives (*m/z* 253.9) in ethyl acetate extract [31]. Compound 7 in jelly palm fruit and compound 10 in fishtail palm fruit yielding [M − H]^−^ ion at *m/z* at 315.0731 and 153.0200 respectively, were tentatively identified as protocatechuic acid 4-*O*-glucoside and protocatechuic acid (Table 2 and Table 3). Protocatechuic acid showing *m/z* 153.2 has already been reported in two açai palm fruits in negative ionization mode [32]. Also, compound 12 in fishtail palm fruit found in positive ionization mode with RT = 42.872 min and [M + H]^+^ at *m/z* 605.0204 was tentatively identified as gallagic acid (Table 3), a very versatile antioxidant with promising therapeutic and industrial applications potential [33]. 

##### Hydroxyphenylacetic, Hydroxyphenylpentanoic and Hydroxyphenylpropanoic Acids

The hydroxyphenylacetic acids and hydroxyphenylpropanoic acids were only characterized in fishtail palm as shown in Table 3. Compound 13 (RT = 22.911 min) showing [M + H]^+^ ion at *m/z* 169.0483 was tentatively identified as 3,4-dihydroxyphenylacetic acid. 2-Hydroxy-2-phenylacetic acid displaying [M − H]^−^ ion at *m/z* 151.0401 was also tentatively characterized in fishtail palm fruits. Compound 17 (RT = 16.401 min) and compound 18 (RT = 21.073 min) giving precursor ion [M + H]^+^ at *m/z* 167.0687 and 227.0916 were assigned as 3-hydroxyphenylpropionic acid and dihydrosinapic acid, while 3-hydroxy-3-(3-hydroxyphenyl) propionic acid was tentatively identified by a molecular ion at *m/z* 181.0513 in negative ionization mode. The hydroxyphenylpentanoic acid compounds conjugated with valerolactone were proposed for both palm fruits (Table 2 and Table 3). 5-(3’-Methoxy-4’-hydroxyphenyl)-γ-valerolactone which exhibited molecular ion at *m/z* 221.0828 was tentatively characterized for jelly palm in ESI− mode (Table 2). In the fishtail palm fruit, 5-(3’,5’-dihydroxyphenyl)-γ-valerolactone 3-*O*-glucuronide ([M + H]^+^ *m/z* 385.1136) and 5-(3’,4’,-dihydroxyphenyl)-γ-valerolactone ([M − H]^−^ *m/z* 207.0667) were detected (Table 3). Previously, there was no report demonstrating the detection of hydroxyphenylacetic, hydroxyphenylpentanoic and hydroxyphenylpropanoic acids in both jelly and fishtail palm fruits despite 3,4-dihydroxyphenylacetic acid reported in ethyl acetate extract of date palm fruits after alkaline processing [31]. 

#### 3.3.2. Flavonoids

Flavonoids are the main class of polyphenols, tentatively identified and characterized by LC-ESI-QTOF/MS in our study. Eight subclasses of flavonoids including flavonols, flavanones, anthocyanins, flavones, isoflavonoids, flavanols, dihydroflavonols and dihydrochalcone were characterized in jelly and fishtail palm fruits as shown in Table 2 and Table 3.

##### Flavonols

Kaempferol glycoside derivatives were tentatively characterized in ethanolic extracts of both jelly and fishtail palm fruits (Table 2 and Table 3). Compound 21 (RT = 25.373 min, *m/z* 755.2044) and 23 (RT = 32.85 min, *m/z* 447.0939) detected in ethanolic extract of fishtail palm fruits were tentatively identified as kaempferol 3-*O*-glucosyl-rhamnosyl-galactoside and kaempferol 3-*O*-glucoside, respectively, in negative ionization mode (Table 3). These derivatives were also observed in the jelly palm, despite kaempferol 3-*O*-glucoside (14) tentatively identified by the parent ion at *m/z* 449.1072 in ESI+ mode (Table 2). Kaempferol 3,7,4’-*O*-triglucoside and kaempferol 3-*O*-xylosyl-glucoside showing parent ions at *m/z* 771.1983 and 581.1472 in ESI and ESI+ modes, respectively were characterized in the jelly palm. Beskow, Hoffmann, Teixeira, Fachinello, Chaves and Rombaldi [6] have indicated the presence of the aglycone kaempferol in ethanolic extract of grounded jelly palm powders. Besides, kaempferol derivatives have been identified in the jelly palm and date palm fruits [21,30]. Some quercetin derivatives were also found in our palm fruits as shown in Table 2 and Table 3. Quercetin 3-*O*-glucoside (15), quercetin 3-*O*-rutinoside (16), and quercetin (17) exhibiting precursor ion at *m/z* 465.1008, 611.1579 and 303.0486 in positive ionization mode were proposed for jelly palm fruit (Table 2). These compounds were also tentatively characterized in fishtail palm fruits despite quercetin 3-*O*-glucoside (RT = 37.133 min) giving precursor ion at *m/z* 463.0878 in ESI− mode (Table 3). Compound 28 with [M + H]^+^ at *m/z* 551.1008 in fishtail palm fruit was characterized as quercetin 3-*O*-(6”-malonyl-glucoside). The detection of aglycone quercetin in the jelly palm was reported previously [3,6,27]. In addition, it was proven that jelly palm presents constituents of quercetin glucosides derivatives and quercetin 3-*O*-rutinoside [3,26]. Quercetin 3-*O*-rutinoside has been found in leaves of fishtail palm by LC-MS analysis in the study of Sujitha and Kripa [20]. Moreover, the quercetin derivative constituents in date palm fruits were also investigated by chromatography analysis [30,34]. 

Compound 29 (RT = 43.141 min) and 30 (RT = 43.332 min) showing [M + H]^+^ ions at *m/z* 625.1746 and 317.0672 were tentatively identified as isorhamnetin 3-*O*-glucoside 7-*O*-rhamnoside and isorhamnetin, respectively, in the fishtail palm. These compounds were also tentatively characterized in jelly palm despite isorhamnetin 3-*O*-glucoside 7-*O*-rhamnoside (18, *m/z* 623.1614) detected in negative ionization mode. Hoffmann, Carvalho, Barbieri, Rombaldi and Chaves [21] also characterized isorhamnetin derivatives with the same molecular formula (C_28_H_32_O_16_ and *m/z* 623.1620) as isorhamnetin 3-*O*-glucoside 7-*O*-rhamnoside in the jelly palm. The glycoside derivatives of isorhamnetin have also been proposed in date palm fruits in previous studies [30,34]. Compound 22 (RT = 30.697 min) and 27 (RT = 37.77 min) in fishtail palm fruit exhibiting [M + H]^+^ ions at 433.1481 and 535.1089 were tentatively identified as 3-methoxynobiletin and 5,4’-dihydroxy-3,3’-dimethoxy-6:7-methylenedioxyflavone 4’-*O*-glucuronide, respectively, while myricetin 3-*O*-rutinoside in jelly palm and 3-methoxysinensetin in the fishtail palm were tentatively characterized in negative ionization mode. Myricetin has been identified in date palm fruits using the high performance liquid chromatography with diode-array detection (HPLC-DAD) in the study of Eid et al. [35].

##### Flavones, Flavanones and Flavanols

Apigenin glucoside derivatives were detected in both palm fruits in the positive mode of ionization as shown in Table 2 and Table 3. Apigenin 6,8-di-C-glucoside displaying a precursor ion at *m/z* 595.1634 and 595.163, respectively, was tentatively characterized in extracts of jelly and fishtail palm fruits. Compound 21 (RT = 29.891 min, *m/z* 565.1523) in the jelly palm and compound 32 (RT = 37.422 min, *m/z* 433.1111) in the fishtail palm were tentatively identified as apigenin 7-*O*-apiosyl-glucoside and apigenin 6-C-glucoside, respectively. The aglycone apigenin has been identified in different solvent extracts of jelly palm [27]. Açai palm fruits also showed the presence of glucoside derivatives of apigenin, including apigenin di-glucosides and apigenin 6-C-glucoside [29,32]. Compound 33 and 34 in fishtail palms showing [M − H]^−^ ion at *m/z* 461.1093 and [M + H]^+^ ion at *m/z* 549.1220, were proposed as chrysoeriol 7-*O*-glucoside and chrysoeriol 7-*O*-(6’’-malonyl-glucoside), respectively (Table 3). Farag, Mohsen, Heinke and Wessjohann [28] also discovered the chrysoeriol compound conjugated with hexoside in the skin of date palms using LC-ESI-QTOF/MS. Compound 22 in jelly palm presenting a molecular ion at *m/z* 607.1674 in ESI mode was tentatively characterized as neodiosmin (Table 2). Hesperetin 3’-*O*-glucuronide, a flavanone constituent exhibiting [M + H]^+^ ion at *m/z* 479.1152 and [M − H]^−^ ion at *m/z* 477.1048 was detected in both jelly (23, RT = 43.01 min) and fishtail palm fruit (36, 42.762 min), respectively. The aglycone hesperetin displaying [M − H]^−^ ion at *m/z* 301.0707 has been proposed for jelly palm fruits in the study of Hoffmann, Carvalho, Barbieri, Rombaldi and Chaves [21]. Other flavanones (compounds 24, 25 and 26) presenting precursor ion at *m/z* 433.1135, 827.2265 and 407.1888 in negative ionization mode were tentatively identified as naringenin 7-*O*-glucoside, naringin 6’-malonate and 6-geranylnaringenin, respectively, in the jelly palm. Boeing et al. [27] characterized the naringenin in extracts of jelly palm. Naringenin methyl ether has been found in date palm peel in the study of Farag, Mohsen, Heinke and Wessjohann [28].

Catechins are a very common flavanol found in most fruits and vegetables. Catechin presenting [M + H]^+^ ion at *m/z* 291.0850 at the retention time of 25.832 min was proposed as compound 28 in the jelly palm. Compound 38 in fishtail palm fruit with [M + H]^+^ ion at *m/z* 481.1340 was proposed as 3’-*O*-methyl-(-)-epicatechin 7-*O*-glucuronide. Catechin has also been detected in ethanolic extracts of jelly palm in a study reported by Beskow, Hoffmann, Teixeira, Fachinello, Chaves and Rombaldi [6]. Procyanidin dimer B1 (RT = 15.131 and 15.613 min) showing the precursor ions at *m/z* 579.1470 and 579.1476 in positive ionization mode were detected in jelly and fishtail palm fruits, respectively. Previously, two compounds of procyanidin dimers with *m/z* 577.1 were detected in *Euterpe oleracea* açai fruits in negative ionization mode [32]. 

##### Anthocyanins, Isoflavonoids, Dihydroflavonols and Dihydrochalcones

The anthocyanins constituents were tentatively identified only in negative ionization mode as shown in Table 2 and Table 3. Peonidin glycoside derivatives were tentatively identified in the ethanolic extract of both jelly and fishtail palm fruits. Peonidin 3-*O*-sambubioside-5-*O*-glucoside (RT = 25.648 min) and peonidin 3-*O*-diglucoside-5-*O*-glucoside (RT = 26.857 min) exhibiting precursor ions at *m/z* 756.2092 and 786.2195 were detected in jelly palm (Table 2), while peonidin 3-*O*-sophoroside (RT = 44.148 min) with parent ions at *m/z* 624.1664 was tentatively characterized in fishtail palm (Table 3). Two compounds in jelly palm showing *m/z* 772.2033 and 564.1457 were assigned as cyanidin 3-*O*-diglucoside-5-*O*-glucoside (29) and pelargonidin 3-*O*-sambubioside (32), respectively. In fishtail palm fruit, compound 40 exhibiting precursor ion at *m/z* 520.1225 was proposed as petunidin 3-*O*-(6’’-acetyl-glucoside). Previously, two cyanidin derivative anthocyanins were characterized in jelly palm fruit [6]. In the study of Pacheco-Palencia, Duncan and Talcott [32], cyanidin derivatives, peonidin derivatives and pelargonidin derivatives were proposed as anthocyanins compounds in açai fruits.

Isoflavonoid constituents were also found in both jelly and fishtail palms as shown in Table 2 and Table 3. Compound 35 (RT = 47.118 min) and 36 (RT = 70.458 min) detected in extract of jelly palm showing [M + H]^+^ ion at *m/z* 273.0757 and 419.1332 were tentatively characterized as 3’,4’,7-trihydroxyisoflavanone and equol 7-*O*-glucuronide (Table 2), while 2-dehydro-*O*-desmethylangolensin detected in jelly palm exhibited precursor ion at *m/z* 255.0673 in ESI− mode. In addition, daidzein 4’-*O*-glucuronide with [M + H]^+^ at *m/z* 431.0956 was proposed for compound 41 in fishtail palm (Table 3). Dihydroflavonols and dihydrochalcones were only tentatively identified in the jelly palm fruits. Compound 33 and 34 at RT = 18.442 and 26.526 min exhibiting molecular ions at *m/z* 465.1038 and 449.1112 in negative ionization mode were proposed as dihydromyricetin 3-*O*-rhamnoside and dihydroquercetin 3-*O*-rhamnoside. Phloretin 2’-*O*-xylosyl-glucoside displaying [M + H]^+^ ion at *m/z* 569.1846 was proposed as dihydrochalcones constituents in jelly palm. Khallouki, Ricarte, Breuer and Owen [30] found the presence of dihydroquercetin 3-*O*-rhamnoside with [M − H]^−^ ion at *m/z* 449.1 in date palm fruits using HPLC-DAD-ESI-MS. To our knowledge, anthocyanin and isoflavonoid compounds have never been reported in fishtail palm fruits in the literature.

#### 3.3.3. Lignans and Stilbenes

As shown in Table 2 and Table 3, seven types of lignans were tentatively identified in the extract of fishtail palm fruit but only one type was identified in jelly palm fruit. Three compounds showing a matairesinol structure were identified in fishtail palm fruit. 7-Oxomatairesinol (RT = 29.769 min), matairesinol (RT = 34.225 min) and dimethylmatairesinol (RT = 81.552 min) showing [M + H]^+^ ion at *m/z* 373.1263, 359.1483 and 387.1793, respectively, were detected in fishtail palm fruit (Table 3). Compounds 43, 44 and 46 in fishtail palm with parent ions at *m/z* 559.2574, 355.1178 and 377.157, respectively, in positive ionization mode were tentatively characterized as secoisolariciresinol-sesquilignan, episesami and todolactol A, while syringaresinol was tentatively identified by parent ion at *m/z* 417.1564 in ESI− mode. In jelly palm fruits, only schisandrin B as lignans constituents with [M + H]^+^ ion at *m/z* 401.1946 was proposed (Table 2). The resveratrol 3-O-glucoside (RT = 39.161 min) as stilbene compound, showing [M − H]^−^ ion at *m/z* 389.1244 was found only in fishtail palm. The lignans constituents in the current study were first reported in jelly and fishtail palm fruits. Previously, date palm fruits showed the presence of lignans compounds in the study of Farag, Mohsen, Heinke and Wessjohann [28].

#### 3.3.4. Other Polyphenols

The LC-ESI-QTOF/MS detected 12 compounds belonging to seven different subclasses of other polyphenols in jelly and fishtail palm fruits (Table 2 and Table 3). Most of these compounds were first reported in jelly and fishtail palm fruits. We divided compounds of other polyphenols into two subgroups: tyrosols and others.

##### Tyrosols

A total of four compounds with a hydroxytryrosol structure were proposed for both jelly and fishtail palm fruits in negative ionization mode (Table 2 and Table 3). Compound 40 (RT = 8.571 min) and 41 (RT = 9.746 min) giving parent ion at *m/z* 153.0555 and 315.1091 in ethanolic extract of jelly palm fruits were tentatively identified as hydroxytyrosol and hydroxytyrosol 4-*O*-glucoside. The presence of hydroxytyrosol, 3,4-DHPEA-AC and demethyloleuropein were found in fishtail palm fruit, while p-HPEA-AC in fishtail palm was tentatively characterized by [M + H]^+^ ion at *m/z* 181.0844 at the retention time of 44.28 min. It is the first time that tyrosols compounds were reported in jelly and fishtail palm fruits.

##### Others

Compound 54 (RT = 33.745 min) and 55 (RT = 79.779 min) tentatively characterized in the extract of fishtail palm fruit with [M − H]^−^ ion at *m/z* 177.0553 and 191.0335 were proposed as hydroxycoumarins compounds mellein and scopoletin (Table 3). 3-Methylcatechol in fishtail palm was tentatively characterized by [M − H]^−^ ion at *m/z* 123.0460 at the retention time of 7.075 min. Compound 57 detected in fishtail palm fruits with [M + H]^+^ ion at *m/z* 339.1239 was tentatively identified as demethoxycurcumin. 2,3-Dihydroxy-1-guaiacylpropanone (compound 58) exhibiting precursor ion at *m/z* 211.0617 in ESI− mode was detected in fishtail palm fruit. Thymol (compound 59) displaying [M + H]^+^ ion at *m/z* 151.1108 was detected in fishtail palm. Pyrogallol was the only compound belonging to other polyphenols subclass detected in both palm fruits in the positive mode of ionization (Table 2 and Table 3). Previously, the presence of hydroxycoumarins compounds (umbelliferone derivatives) was reported in fishtail palm fruits [20]. Patel et al. [36] indicated the possible presence of thymol in the leaf of fishtail palm after chromatography and spectroscopic analysis. The concentrated methanolic extract of immature fishtail palm fruits highlighted the pyrogallol 1,3-dimethyl ether constituents [9].

The screening and characterization of polyphenolic compounds showed that some of the polyphenols presented in two palm fruits have strong antioxidant potential. Hydroxycinnamic acid derivatives, hydroxybenzoic acids and their derivatives, protocatechuic acid, chlorogenic acid, catechin, matairesinol, hydroxytyrosol, quercetin and kaempferol derivatives are regarded as potential compounds showing considerable free radical scavenging capacity [37,38,39]. The presence of these antioxidant compounds indicates that both jelly and fishtail palm fruits can be good sources of polyphenols and antioxidant potential. 

### 3.4. Quantitative Analysis of Phenolic Compounds by HPLC-PDA

Seven polyphenols were targeted to quantify through HPLC-PDA including three phenolic acids (chlorogenic acid, protocatechuic acid and 4-hydroxybenzoic acid) and four flavonoids (quercetin 3-*O*-glucoside, catechin, quercetin and kaempferol 3-*O*-glucoside) based on the LC-ESI-QTOF/MS characterization and previously reported antioxidant activities. The quantification of individual polyphenols was computed considering the UV–Vis absorption and calibration curves of external standards.

The polyphenols quantified through HPLC-PDA in the jelly palm were higher than in fishtail palm. These HPLC results support our TPC values determined using the Folin–Ciocalteu method. Among the three selected phenolic acids, chlorogenic acid was the only phenolic acid detected in both palm fruits (Table 4). The concentration of chlorogenic acid in the jelly palm (290.10 ± 3.98 µg/g _fw_) was higher than fishtail palm fruit (140.23 ± 1.78 µg/g _fw_). Boeing et al. [27] already reported the chlorogenic acid content in another jelly palm species. Moreover, 4-hydroxybenzoic acid (RT = 25.337 min, 317.46 ± 4.68 µg/g _fw_) was only detected in jelly palm fruit, while protocatechuic acid (RT = 6.929 min, 68.46 ± 0.97 µg/g _fw_) was only found in fishtail palm. Beskow, Hoffmann, Teixeira, Fachinello, Chaves and Rombaldi [6] also quantified hydroxybenzoic acid content with an average value of 123.39 mg/100g in five different genotypes of jelly palm using HPLC.

Flavonols were found to be higher in fishtail palm fruit as compared to jelly palm fruits. Quercetin was the major compound in this group, with a concentration of 360.19 ± 4.53 µg/g _fw_ in jelly palm and 557.28 ± 7.81 µg/g _fw_ in fishtail palm fruit. Beskow, Hoffmann, Teixeira, Fachinello, Chaves and Rombaldi [6] already reported higher quercetin contents in Berzelian jelly palm. Quercetin 3-O-glucoside and kaempferol 3-O-glucoside were also present in both palm fruits. The quercetin and kaempferol 3-O-glucoside content might account for the higher TFC value in fishtail palm fruit as compared to jelly palm. In our results, we found that catechin was high in jelly palm fruit (4.72 ± 0.03 mg/g _fw_), which might be involved in higher antioxidant activity.

## 4. Conclusions 

It has been established in this work that LC-ESI-QTOF/MS is an effective and powerful analytical tool to characterize most of the polyphenols present in jelly and fishtail palm fruits. The TPC, TTC, DPPH, FRAP and ABTS scavenging activity was higher in jelly palm compared to fishtail palm. LC-ESI-QTOF/MS characterizes a total of 42 and 60 phenolic compounds in jelly and fishtail palm fruits, respectively. Hydroxycinnamic acids and flavonols were the most common polyphenols reported in both palm fruits. The HPLC-PDA enabled the quantification of some targeted phenolic compounds and found that catechin and quercetin were the most abundant polyphenols in jelly and fishtail palm, respectively. In short, both palm fruits are a good source of polyphenols and could be utilized in food, feed and pharmaceutical industries.

## Figures and Tables

**Table 1:** Total phenolic content (TPC), total flavonoid content (TFC) and total tannins content (TTC) and antioxidant activities (2,2-diphenyl-1-picrylhydrazyl (DPPH), ferric reducing antioxidant power (FRAP) and 2,2′-azinobis-(3-ethylbenzo-thiazoline-6-sulfonic acid (ABTS)) of palm fruit samples.

Antioxidant Assays	Jelly Palm	Fishtail Palm
TPC (mg GAE/g)	3.74 ± 0.10 ^a^	1.75 ± 0.02 ^b^
TFC (µg QE/g)	63.11 ± 0.51 ^b^	207.80 ± 4.8 ^a^
TTC (mg CE/g)	4.06 ± 0.04 ^a^	1.02 ± 0.01 ^b^
DPPH (mg AAE/g)	2.58 ± 0.06 ^a^	0.31 ± 0.01 ^b^
FRAP (mg AAE/g)	1.29 ± 0.01 ^a^	0.03 ± 0.01 ^b^
ABTS (mg AAE/g)	4.38 ± 0.05 ^a^	1.85 ± 0.04 ^b^

The data are shown as mean ± standard deviation (*n* = 3); ^a,b^ indicate the means in a row with significant difference (*p* < 0.05) using a one-way analysis of variance (ANOVA) and Tukey’s test. GAE, gallic acid equivalents; QE, quercetin equivalents; CE, catechin equivalents; AAE, ascorbic acid equivalents.

**Table 2:** Liquid chromatography–electrospray ionization quadrupole time-of-flight mass spectrometry (LC-ESI-QTOF/MS) characterization of jelly palm fruit polyphenolic compounds. RT, retention time.

Peak No.	Proposed Compound	Molecular Formula	RT (min)	Ionization Mode	Molecular Weight	Theoretical (*m/z*)	Observed (*m/z*)	Mass Error (ppm)
**Phenolic Acid**								
**Hydroxycinnamic Acids**								
1	Cinnamic acid	C_9_H_8_O_2_	9.201	ESI+/[M + H]^+^	148.0524	149.0597	149.0585	–8.05
2	Chlorogenic acid	C_16_H_18_O_9_	19.734	ESI−/[M − H]^−^	354.0951	353.0878	353.0890	3.4
3	Ferulic acid 4-*O*-glucoside	C_16_H_20_O_9_	26.043	ESI−/[M − H]^−^	356.1107	355.1034	355.1049	4.22
4	3-*p*-Coumaroylquinic acid	C_16_H_18_O_8_	27.074	ESI+/[M + H]^+^	338.1002	339.1075	339.1081	1.77
5	1,5-Dicaffeoylquinic acid	C_25_H_24_O_12_	37.691	ESI−/[M − H]^−^	516.1268	515.1195	515.1187	–1.55
6	1-Sinapoyl-2-feruloylgentiobiose	C_33_H_40_O_18_	41.501	ESI−/[M − H]^−^	724.2215	723.2142	723.2137	–0.69
**Hydroxybenzoic Acids**								
7	Protocatechuic acid 4-*O*-glucoside	C_13_H_16_O_9_	9.166	ESI−/[M − H]^−^	316.0794	315.0721	315.0731	3.17
8	4-Hydroxybenzoic acid	C_7_H_6_O_3_	25.849	ESI+/[M + H]^+^	138.0317	139.0390	139.0394	2.88
**Hydroxyphenylpentanoic Acids**								
9	5-(3’-Methoxy-4’-hydroxyphenyl)-γ-valerolactone	C_12_H_14_O_4_	25.781	ESI−/[M − H]^−^	222.0892	221.0819	221.0828	4.07
**Flavonoids**								
**Flavonols**								
10	Kaempferol 3,7,4’-*O*-triglucoside	C_33_H_40_O_21_	20.745	ESI−/[M − H]^−^	772.2062	771.1989	771.1983	–0.78
11	Myricetin 3-*O*-rutinoside	C_27_H_30_O_17_	21.209	ESI−/[M − H]^−^	626.1483	625.1410	625.1412	0.32
12	Kaempferol 3-*O*-glucosyl-rhamnosyl-galactoside	C_33_H_40_O_20_	25.648	ESI−/[M − H]^−^	756.2113	755.2040	755.2056	2.12
13	Kaempferol 3-*O*-xylosyl-glucoside	C_26_H_28_O_15_	27.952	ESI+/[M + H]^+^	580.1428	581.1501	581.1472	–4.99
14	Kaempferol 3-*O*-glucoside	C_21_H_20_O_11_	33.181	ESI+/[M + H]^+^	448.1006	449.1079	449.1072	–1.56
15	Quercetin 3-*O*-glucoside	C_21_H_20_O_12_	37.196	ESI+/[M + H]^+^	464.0955	465.1028	465.1008	–4.3
16	Quercetin 3-*O*-rutinoside	C_27_H_30_O_16_	37.196	ESI+/[M + H]^+^	610.1534	611.1607	611.1579	–4.58
17	Quercetin	C_15_H_10_O_7_	37.462	ESI+/[M + H]^+^	302.0427	303.0500	303.0486	–4.62
18	Isorhamnetin 3-*O*-glucoside 7-*O*-rhamnoside	C_28_H_32_O_16_	42.892	ESI−/[M − H]^−^	624.1690	623.1617	623.1614	–0.48
19	Isorhamnetin	C_16_H_12_O_7_	43.043	ESI+/[M + H]^+^	316.0583	317.0656	317.0637	–5.99
**Flavones**								
20	Apigenin 6,8-di-C-glucoside	C_27_H_30_O_15_	27.164	ESI+/[M + H]^+^	594.1585	595.1658	595.1634	–4.03
21	Apigenin 7-*O*-apiosyl-glucoside	C_26_H_28_O_14_	29.891	ESI+/[M + H]^+^	564.1479	565.1552	565.1523	–5.13
22	Neodiosmin	C_28_H_32_O_15_	44.731	ESI−/[M − H]^−^	608.1741	607.1668	607.1674	0.98
**Flavanones**								
23	Hesperetin 3’-*O*-glucuronide	C_22_H_22_O_12_	43.01	ESI+/[M + H]^+^	478.1111	479.1184	479.1152	–6.68
24	Naringenin 7-*O*-glucoside	C_21_H_22_O_10_	47.083	ESI−/[M − H]^−^	434.1213	433.1140	433.1135	–1.15
25	Naringin 6’-malonate	C_36_H_44_O_22_	61.396	ESI−/[M − H]^−^	828.2324	827.2251	827.2265	1.69
26	6-Geranylnaringenin	C_25_H_28_O_5_	79.899	ESI−/[M − H]^−^	408.1937	407.1864	407.1888	5.89
**Flavanols**								
27	Procyanidin dimer B1	C_30_H_26_O_12_	15.131	ESI+/[M + H]^+^	578.1424	579.1497	579.1470	-4.66
28	(+)-Catechin	C_15_H_14_O_6_	25.832	ESI+/[M + H]^+^	290.0790	291.0863	291.0850	–4.47
**Anthocyanins**								
29	Cyanidin 3-*O*-diglucoside-5-*O*-glucoside	C_33_H_41_O_21_	21.606	ESI−/[M − H]^−^	773.2140	772.2067	772.2033	–4.4
30	Peonidin 3-*O*-sambubioside-5-*O*-glucoside	C_33_H_41_O_20_	25.648	ESI−/[M − H]^−^	757.2191	756.2118	756.2092	–3.44
31	Peonidin 3-*O*-diglucoside-5-*O*-glucoside	C_34_H_43_O_21_	26.857	ESI−/[M − H]^−^	787.2297	786.2224	786.2195	–3.69
32	Pelargonidin 3-*O*-sambubioside	C_26_H_29_O_14_	31.098	ESI−/[M − H]^−^	565.1557	564.1484	564.1457	–4.79
**Dihydroflavonols**								
33	Dihydromyricetin 3-*O*-rhamnoside	C_21_H_22_O_12_	18.442	ESI−/[M − H]^−^	466.1111	465.1038	465.1038	0
34	Dihydroquercetin 3-*O*-rhamnoside	C_21_H_22_O_11_	26.526	ESI−/[M − H]^−^	450.1162	449.1089	449.1112	5.12
**Isoflavonoids**								
35	3’,4’,7-Trihydroxyisoflavanone	C_15_H_12_O_5_	47.118	ESI+/[M + H]^+^	272.0685	273.0758	273.0757	–0.37
36	Equol 7-*O*-glucuronide	C_21_H_22_O_9_	70.458	ESI+/[M + H]^+^	418.1264	419.1337	419.1332	–1.19
37	2-Dehydro-*O*-desmethylangolensin	C_15_H_12_O_4_	77.232	ESI−/[M − H]^−^	256.0736	255.0663	255.0673	3.92
**Dihydrochalcones**								
38	Phloretin 2’-*O*-xylosyl-glucoside	C_26_H_32_O_14_	46.075	ESI+/[M + H]^+^	568.1792	569.1865	569.1846	–3.34
**Lignans**								
**Lignans**								
39	Schisandrin B	C_23_H_28_O_6_	80.779	ESI+/[M + H]^+^	400.1886	401.1959	401.1946	–3.24
**Other Polyphenols Tyrosols**								
40	Hydroxytyrosol	C_8_H_10_O_3_	8.571	ESI−/[M − H]^−^	154.0630	153.0557	153.0555	–1.31
41	Hydroxytyrosol 4-*O*-glucoside	C_14_H_20_O_8_	9.746	ESI−/[M − H]^−^	316.1158	315.1085	315.1091	1.9
**Other Polyphenols**								
42	Pyrogallol	C_6_H_6_O_3_	10.743	ESI+/[M + H]^+^	126.0317	127.0390	127.0384	–4.72

**Table 3:** LC-ESI-QTOF/MS characterization of fishtail palm fruit polyphenolic compounds.

Peak No.	Proposed Compound	Molecular Formula	RT (min)	Ionization Mode	Molecular Weight	Theoretical (*m/z*)	Observed (*m/z*)	Mass Error (ppm)
**Phenolic Acids**								
**Hydroxycinnamic Acids**								
1	Cinnamic acid	C_9_H_8_O_2_	9.474	ESI+/[M + H]^+^	148.0524	149.0597	149.0582	–10.06
2	Chlorogenic acid	C_16_H_18_O_9_	19.979	ESI+/[M + H]^+^	354.0951	355.1024	355.1017	–1.97
3	Sinapine	C_16_H_24_NO_5_	24.568	ESI−/[M − H]^−^	310.1654	309.1581	309.1568	–4.20
4	Ferulic acid 4-*O*-glucoside	C_16_H_20_O_9_	25.81	ESI−/[M − H]^−^	356.1107	355.1034	355.1049	4.22
5	3-Feruloylquinic acid	C_17_H_20_O_9_	30.1	ESI−/[M − H]^−^	368.1107	367.1034	367.1040	1.63
6	3-*O*-Methylrosmarinic acid	C_19_H_18_O_8_	31.227	ESI+/[M + H]^+^	374.1002	375.1075	375.1069	–1.60
7	Ferulic acid	C_10_H_10_O_4_	37.919	ESI−/[M − H]^−^	194.0579	193.0506	193.0504	–1.04
8	1-Sinapoyl-2-feruloylgentiobiose	C_33_H_40_O_18_	41.78	ESI−/[M − H]^−^	724.2215	723.2142	723.2167	3.46
9	1,5-Diferuloylquinic acid	C_27_H_28_O_12_	63.86	ESI−/[M − H]^−^	544.1581	543.1508	543.1529	3.87
**Hydroxybenzoic Acids**								
10	Protocatechuic acid	C_7_H_6_O_4_	7.158	ESI−/[M − H]^−^	154.0266	153.0193	153.0200	4.57
11	4-Hydroxybenzoic acid 4-*O*-glucoside	C_13_H_16_O_8_	7.456	ESI−/[M − H]^−^	300.0845	299.0772	299.0793	7.02
12	Gallagic acid	C_28_H_12_O_16_	42.872	ESI+/[M + H]^+^	604.0125	605.0198	605.0204	0.99
**Hydroxyphenylacetic Acids**								
13	3,4-Dihydroxyphenylacetic acid	C_8_H_8_O_4_	22.911	ESI+/[M + H]^+^	168.0423	169.0496	169.0483	–7.69
14	2-Hydroxy-2-phenylacetic acid	C_8_H_8_O_3_	31.84	ESI−/[M − H]^−^	152.0473	151.0400	151.0401	0.66
**Hydroxyphenylpentanoic Acids**								
15	5-(3’,5’-dihydroxyphenyl)-γ-valerolactone 3-*O*-glucuronide	C_17_H_20_O_10_	10.156	ESI+/[M + H]^+^	384.1056	385.1129	385.1136	1.82
16	5-(3’,4’,-dihydroxyphenyl)-γ-valerolactone	C_11_H_12_O_4_	68.647	ESI−/[M − H]^−^	208.0736	207.0663	207.0667	1.93
**Hydroxyphenylpropanoic Acids**								
17	3-Hydroxyphenylpropionic acid	C_9_H_10_O_3_	16.401	ESI+/[M + H]^+^	166.0630	167.0703	167.0687	–9.58
18	Dihydrosinapic acid	C_11_H_14_O_5_	21.073	ESI+/[M + H]^+^	226.0841	227.0914	227.0916	0.88
19	3-Hydroxy-3-(3-hydroxyphenyl) propionic acid	C_9_H_10_O_4_	33.927	ESI−/[M − H]^−^	182.0579	181.0506	181.0513	3.87
**Flavonoids**								
**Flavonols**								
20	3-Methoxysinensetin	C_21_H_22_O_8_	17.975	ESI−/[M − H]^−^	402.1315	401.1242	401.1240	–0.50
21	Kaempferol 3-*O*-glucosyl-rhamnosyl-galactoside	C_33_H_40_O_20_	25.373	ESI−/[M − H]^−^	756.2113	755.2040	755.2044	0.53
22	3-Methoxynobiletin	C_22_H_24_O_9_	30.697	ESI+/[M + H]^+^	432.1420	433.1493	433.1481	–2.77
23	Kaempferol 3-*O*-glucoside	C_21_H_20_O_11_	32.85	ESI−/[M − H]^−^	448.1006	447.0933	447.0939	1.34
24	Quercetin 3-*O*-rutinoside	C_27_H_30_O_16_	36.831	ESI+/[M + H]^+^	610.1534	611.1607	611.1586	–3.44
25	Quercetin 3-*O*-glucoside	C_21_H_20_O_12_	37.133	ESI−/[M − H]^−^	464.0955	463.0882	463.0878	–0.86
26	Quercetin	C_15_H_10_O_7_	37.594	ESI+/[M + H]^+^	302.0427	303.0500	303.0486	–4.62
27	5,4’-Dihydroxy-3,3’-dimethoxy-6:7-methylenedioxyflavone 4’-*O*-glucuronide	C_24_H_22_O_14_	37.77	ESI+/[M + H]^+^	534.1010	535.1083	535.1089	1.12
28	Quercetin 3-*O*-(6”-malonyl-glucoside)	C_24_H_22_O_15_	42.872	ESI+/[M + H]^+^	550.0959	551.1032	551.1008	–4.35
29	Isorhamnetin 3-*O*-glucoside 7-*O*-rhamnoside	C_28_H_32_O_16_	43.141	ESI+/[M + H]^+^	624.1690	625.1763	625.1746	–2.72
30	Isorhamnetin	C_16_H_12_O_7_	43.332	ESI+/[M + H]^+^	316.0583	317.0656	317.0672	5.05
**Flavones**								
31	Apigenin 6,8-di-C-glucoside	C_27_H_30_O_15_	26.87	ESI+/[M + H]^+^	594.1585	595.1658	595.1630	–4.70
32	Apigenin 6-C-glucoside	C_21_H_20_O_10_	37.422	ESI+/[M + H]^+^	432.1056	433.1129	433.1111	–4.16
33	Chrysoeriol 7-*O*-glucoside	C_22_H_22_O_11_	39.311	ESI−/[M − H]^−^	462.1162	461.1089	461.1093	0.87
34	Chrysoeriol 7-*O*-(6’’-malonyl-glucoside)	C_25_H_24_O_14_	59.653	ESI+/[M + H]^+^	548.1166	549.1239	549.1220	–3.46
**Flavanones**								
35	Eriocitrin	C_27_H_32_O_15_	28.03	ESI+/[M + H]^+^	596.1741	597.1814	597.1822	1.34
36	Hesperetin 3’-*O*-glucuronide	C_22_H_22_O_12_	42.762	ESI−/[M − H]^−^	478.1111	477.1038	477.1048	2.10
**Flavanols**								
37	Procyanidin dimer B1	C_30_H_26_O_12_	15.613	ESI+/[M + H]^+^	578.1424	579.1497	579.1476	–3.63
38	3’-*O*-Methyl-(-)-epicatechin 7-*O*-glucuronide	C_22_H_24_O_12_	28.013	ESI+/[M + H]^+^	480.1268	481.1341	481.1340	–0.21
**Anthocyanins**								
39	Peonidin 3-*O*-sophoroside	C_28_H_33_O_16_	44.148	ESI−/[M − H]^−^	625.1769	624.1696	624.1664	–5.13
40	Petunidin 3-*O*-(6’’-acetyl-glucoside)	C_24_H_25_O_13_	53.159	ESI−/[M − H]^−^	521.1295	520.1222	520.1225	0.58
**Isoflavonoids**								
41	Daidzein 4’-*O*-glucuronide	C_21_H_18_O_10_	31.906	ESI+/[M + H]^+^	430.0900	431.0973	431.0956	–3.94
**Lignans**								
**Lignans**								
42	7-Oxomatairesinol	C_20_H_20_O_7_	29.769	ESI+/[M + H]^+^	372.1209	373.1282	373.1263	–5.09
43	Secoisolariciresinol-sesquilignan	C_30_H_38_O_10_	30.896	ESI+/[M + H]^+^	558.2465	559.2538	559.2574	6.44
44	Episesamin	C_20_H_18_O_6_	31.409	ESI+/[M + H]^+^	354.1103	355.1176	355.1178	0.56
45	Matairesinol	C_20_H_22_O_6_	34.225	ESI+/[M + H]^+^	358.1416	359.1489	359.1483	–1.67
46	Todolactol A	C_20_H_24_O_7_	38.068	ESI+/[M + H]^+^	376.1522	377.1595	377.1570	–6.63
47	Syringaresinol	C_22_H_26_O_8_	64.191	ESI−/[M − H]^−^	418.1628	417.1555	417.1564	2.16
48	Dimethylmatairesinol	C_22_H_26_O_6_	81.552	ESI+/[M + H]^+^	386.1729	387.1802	387.1793	–2.32
**Stilbenes**								
**Stilbenes**								
49	Resveratrol 3-*O*-glucoside	C_20_H_22_O_8_	39.161	ESI−/[M − H]^−^	390.1315	389.1242	389.1244	0.51
**Other Polyphenols Tyrosols**								
50	Hydroxytyrosol	C_8_H_10_O_3_	8.352	ESI−/[M − H]^−^	154.0630	153.0557	153.0571	9.15
51	3,4-DHPEA-AC	C_10_H_12_O_4_	23.292	ESI−/[M − H]^−^	196.0736	195.0663	195.0673	5.13
52	Demethyloleuropein	C_24_H_30_O_13_	26.539	ESI−/[M − H]^−^	526.1686	525.1613	525.1620	1.33
53	p-HPEA-AC	C_10_H_12_O_3_	44.28	ESI+/[M + H]^+^	180.0786	181.0859	181.0844	–8.28
**Hydroxycoumarins**								
54	Mellein	C_10_H_10_O_3_	33.745	ESI−/[M − H]^−^	178.0630	177.0557	177.0553	–2.26
55	Scopoletin	C_10_H_8_O_4_	79.779	ESI−/[M − H]^−^	192.0423	191.0350	191.0335	–7.85
**Alkylphenols**								
56	3-Methylcatechol	C_7_H_8_O_2_	7.075	ESI−/[M − H]^−^	124.0524	123.0451	123.0460	7.31
**Curcuminoids**								
57	Demethoxycurcumin	C_20_H_18_O_5_	9.527	ESI+/[M + H]^+^	338.1154	339.1227	339.1239	3.54
**Hydroxybenzoketones**								
58	2,3-Dihydroxy-1-guaiacylpropanone	C_10_H_12_O_5_	28.676	ESI−/[M − H]^−^	212.0685	211.0612	211.0617	2.37
**Phenolic Terpenes**								
59	Thymol	C_10_H_14_O	39.443	ESI+/[M + H]^+^	150.1045	151.1118	151.1108	–6.62
**Other Polyphenols**								
60	Pyrogallol	C_6_H_6_O_3_	10.504	ESI+/[M + H]^+^	126.0317	127.0390	127.0381	–7.08

**Table 4:** Quantification of polyphenolic compounds in palm samples by high performance liquid chromatography photodiode array (HPLC-PDA).

Compound	Retention Time (min)	Jelly Palm (µg/g)	Fishtail Palm (µg/g)	Polyphenol Class
Protocatechuic acid	6.929	-	68.46 ± 0.97	Phenolic acid
Chlorogenic acid	20.171	290.10 ± 3.98	140.23 ± 1.78	Phenolic acid
4-Hydroxybenzoic acid	25.337	317.46 ± 4.68	-	Phenolic acid
Catechin	26.174	4724.00 ± 32.39	-	Flavonoid
Quercetin 3-*O*-glucoside	37.362	21.47 ± 0.17	97.73 ± 0.91	Flavonoid
Quercetin	37.906	360.19 ± 4.53	557.28 ± 7.81	Flavonoid
Kaempferol 3-*O*-glucoside	33.045	6.14 ± 0.04	220.99 ± 2.06	Flavonoid

## Supplementary Materials

The following are available online at https://www.mdpi.com/2076-3921/8/10/483/s1, Figure S1: LC-ESI-QTOF/MS basic peak chromatograph (BPC) for characterization of phenolic compounds in jelly and fishtail palm fruits. (a) The BPC of jelly palm in negative ionization mode; (b) The BPC of jelly palm in positive ionization mode; (c) The BPC of fishtail palm in negative ionization mode; (d) The BPC of fishtail palm in positive mode, Figure S2: LC-ESI-QTOF/MS identification and characterization of polypheols. (a) A chromatograph of kaempferol 3, 7, 4’ -O-triglucoside (Compound 10, Jelly palm—Table 2), Retention time (RT = 20.745) in the negative mode of ionization (ESI-/[M − H]-); (b) Mass spectra of kaempferol 3, 7, 4’ –Otriglucoside showing an observed m/z 771.1983; (c) A chromatograph of cyanidin 3-O-diglucoside-5-O-glucoside (Compound 29, Jelly palm—Table 2), Retention time (RT = 21.605) in the negative mode of ionization (ESI-/[M − H]−); (d) mass spectra of cyanidin 3-O-diglucoside-5-O-glucoside showing an observed m/z 772.2033.
