# Supplementary material for: LC-ESI-QTOF/MS Characterization of Phenolic Compounds in Palm Fruits (Jelly and Fishtail Palm) and Their Potential Antioxidant Activities
Source: Antioxidants (Basel). 2019 Oct 14;8(10):483. doi: 10.3390/antiox8100483 (PMC6827156; doi:10.3390/antiox8100483)
Supplement: Supplementary file 1 [file antioxidants-08-00483-s001.pdf]

Supplemental material

LC-ESI-QTOF/MS Characterization of Phenolic Compounds in Palm Fruits (Jelly and Fishtail Palm) and Their Potential Antioxidant Activities

Chao Ma, Frank R. Dunshea and Hafiz A. R. Suleria*

School of Agriculture and Food, Faculty of Veterinary and Agricultural Sciences, The University of Melbourne, Parkville, VIC 3010, Australia; chaom2@student.unimelb.edu.au (C.M.); fdunshea@unimelb.edu.au (F.R.D)

* Correspondence: hafiz.suleria@unimelb.edu.au; Tel.: +61-470-439-670

Received: 23 September 2019; Accepted: 10 October 2019; Published: date

Abstract: Palm fruits have gained growing attention for their nutrition values and health promotion perspectives. They have a diverse range of bioactive compounds including carotenoids, vitamins, dietary fibres and especially polyphenolic compounds. These polyphenolic compounds contribute to the putative health benefits of palm fruits. Nevertheless, the detailed information about these polyphenols in palm fruits is limited. The present work was conducted to comprehensively characterize polyphenols in two palm fruits, jelly palm (*Butia ordorata*) and fishtail palm (*Caryota uren*), using liquid chromatography electrospray ionization quadrupole time-of-flight mass spectrometry (LC-ESI-QTOF/MS) and assess their antioxidant potential. The total phenolic content (TPC), total tannins content (TTC), 2,2-diphenyl-1-picrylhydrazyl (DPPH) antioxidant assay and 2,2′-azinobis-(3-ethylbenzo-thiazoline-6-sulfonic acid) (ABTS) scavenging abilities and ferric reducing antioxidant power (FRAP) were higher in the jelly palm fruit while total flavonoid contents (TFC) were higher in the fishtail palm. The LC-ESI-QTOF/MS tentatively identified a total of 86 phenolic compounds in both jelly and fishtail palm fruits. Although both palm fruits exhibited different phenolic profiles, hydroxycinnamic acids and flavonols were the most common in both. In high performance liquid chromatography photodiode array (HPLC-PDA) quantification, 4-hydroxybenzoic acid (317.46 ± 4.68 μg/g) and catechin (4724.00 ± 32.39 μg/g) were the most abundant phenolic acid and flavonoid quantified in the jelly palm fruit, respectively. Quercetin (557.28 ± 7.81 μg/g) and kaempferol 3-*O*-glucoside (220.99 ± 2.06 μg/g) were the most abundant flavonoids quantified in the fishtail palm. Our study indicates that palm fruit is a good source of polyphenols and has strong antioxidant potential for health promotion. Furthermore, this study provides the scientific basis for an exploitation of jelly and fishtail palm fruits in the food, pharmaceutical and nutraceutical industries.

Keywords: jelly palm; fishtail palm; polyphenols; flavonoid; phenolic acids; antioxidant activity; HPLC-PDA; LC-ESI-QTOF/MS

(a)

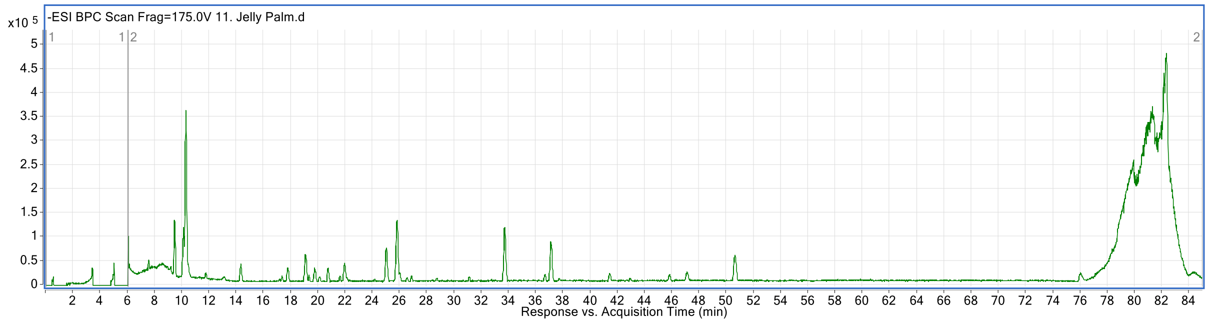

(b)

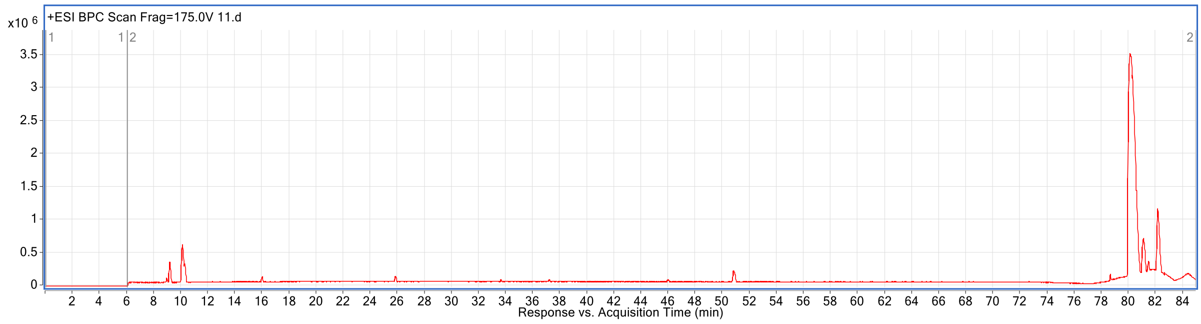

(c)

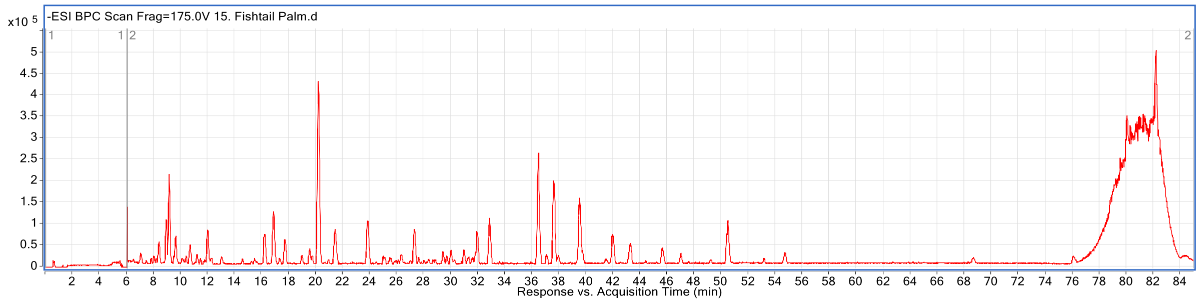

(d)

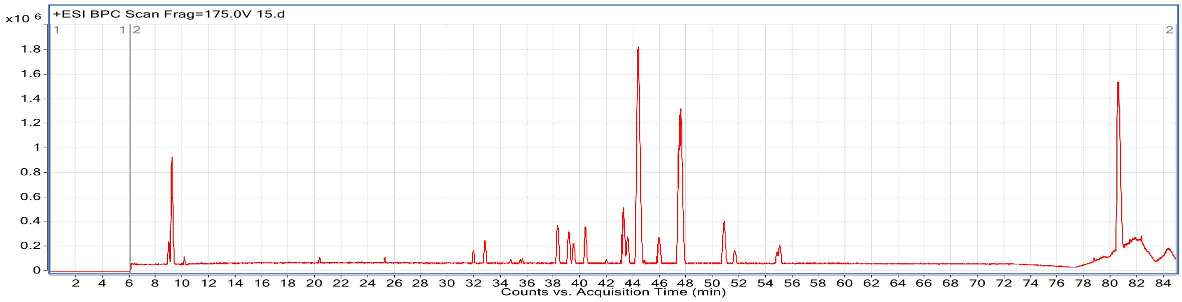

Figure S1. LC-ESI-QTOF/MS basic peak chromatograph (BPC) for characterization of phenolic compounds in jelly and fishtail palm fruits. (a) The BPC of jelly palm in negative ionization mode; (b) The BPC of jelly palm in positive ionization mode; (c) The BPC of fishtail palm in negative ionization mode; (d) The BPC of fishtail palm in positive mode.

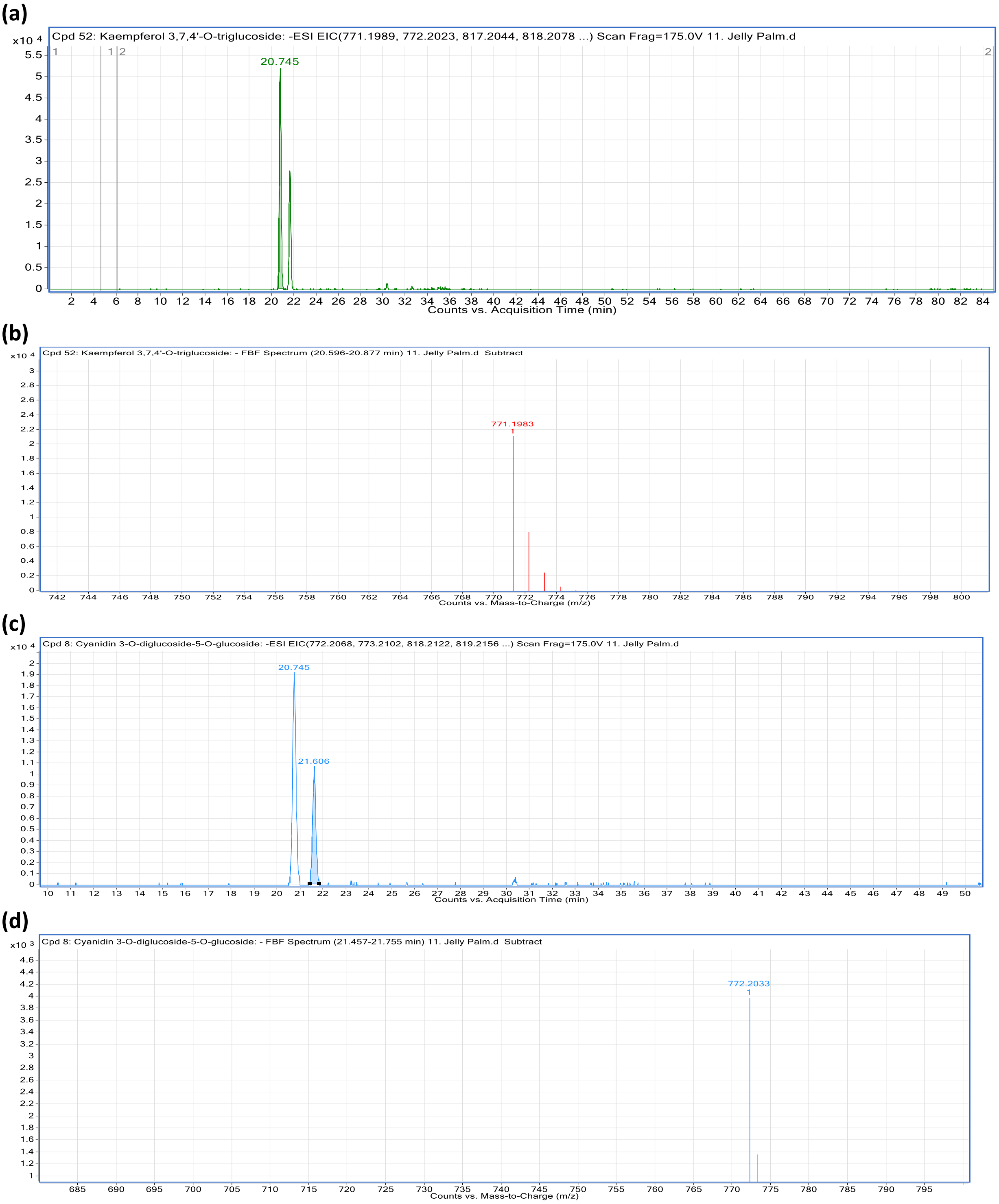

Figure S2. LC-ESI-QTOF/MS identification and characterization of polypheols. (a) A chromatograph of kaempferol 3, 7, 4’ -O-triglucoside (Compound 10, Jelly palm - Table 2), Retention time (RT = 20.745) in the negative mode of ionization (ESI-/$[M-H]^-$); (b) Mass spectra of kaempferol 3, 7, 4’ -O-triglucoside showing an observed *m/z* 771.1983; (c) A chromatograph of cyanidin 3-O-diglucoside-5-O-glucoside (Compound 29, Jelly palm - Table 2), Retention time (RT = 21.605) in the negative mode of ionization (ESI-/$[M-H]^-$); (d) mass spectra of cyanidin 3-O-diglucoside-5-O-glucoside showing an observed *m/z* 772.2033.
